# Inflammatory Bowel Disease: An Overview of Immune Mechanisms and Biological Treatments

**DOI:** 10.1155/2015/493012

**Published:** 2015-08-03

**Authors:** Bruno Rafael Ramos de Mattos, Maellin Pereira Gracindo Garcia, Julia Bier Nogueira, Lisiery Negrini Paiatto, Cassia Galdino Albuquerque, Caique Lopes Souza, Luís Gustavo Romani Fernandes, Wirla Maria da Silva Cunha Tamashiro, Patricia Ucelli Simioni

**Affiliations:** ^1^Department of Genetics, Evolution and Bioagents, Institute of Biology, University of Campinas (UNICAMP), 13083-970 Campinas, SP, Brazil; ^2^Department of Biomedical Science, Faculdade de Americana, Avenida Joaquim Boer 733, 13477-360 Americana, SP, Brazil; ^3^Medical School, UNICAMP, 13083-887 Campinas, SP, Brazil; ^4^Department of Biochemistry and Microbiology, Institute of Biosciences, Universidade Estadual Paulista (UNESP), Avenida 24 A 1515, 13506-900 Rio Claro, SP, Brazil

## Abstract

Inflammatory bowel diseases (IBD) are characterized by chronic inflammation of the intestinal tract associated with an imbalance of the intestinal microbiota. Crohn's disease (CD) and ulcerative colitis (UC) are the most widely known types of IBD and have been the focus of attention due to their increasing incidence. Recent studies have pointed out genes associated with IBD susceptibility that, together with environment factors, may contribute to the outcome of the disease. In ulcerative colitis, there are several therapies available, depending on the stage of the disease. Aminosalicylates, corticosteroids, and cyclosporine are used to treat mild, moderate, and severe disease, respectively. In Crohn's disease, drug choices are dependent on both location and behavior of the disease. Nowadays, advances in treatments for IBD have included biological therapies, based mainly on monoclonal antibodies or fusion proteins, such as anti-TNF drugs. Notwithstanding the high cost involved, these biological therapies show a high index of remission, enabling a significant reduction in cases of surgery and hospitalization. Furthermore, migration inhibitors and new cytokine blockers are also a promising alternative for treating patients with IBD. In this review, an analysis of literature data on biological treatments for IBD is approached, with the main focus on therapies based on emerging recombinant biomolecules.

## 1. Introduction

The role of intestinal milieu in immune homeostasis appears to be of greater significance than it was previously thought. This complex interplay of genetic, microbial, and environmental factors culminates in a sustained activation of the mucosal immune and nonimmune responses. Under normal situations, the intestinal mucosa is in a state of “controlled” inflammation regulated by a delicate balance of Th1, Th17, Th2, Th3, Th9, and Treg cells [1–6].

Inflammatory bowel diseases (IBD) are related to an immunological imbalance of the intestinal mucosa, mainly associated with cells of the adaptive immune system, which respond against self-antigens producing chronic inflammatory conditions in these patients. Ulcerative colitis (UC) and Crohn's disease (CD) are the most studied types of inflammatory bowel diseases, having the highest prevalence in the world population. The pathophysiological mechanisms of IBD are not fully understood, although these diseases have been discovered several decades ago [7–10]. In the present work, we aim to review the current approaches for treating IBD, focusing on the new therapies based on biological molecules.

## 2. Inflammatory Bowel Disease

It is widely known that the number of bacteria in the gastrointestinal tract is about 10 times higher when compared to eukaryotic cells in the body. Also, the normal enteric bacterial flora is a complex ecosystem of approximately 300–500 bacterial species [11, 12]. Moreover, the balance of the innate and adaptive immunity is critical for this microenvironment homeostasis. In this sense, the immune system has the important role of promoting immune tolerance, thereby avoiding the specific immune response against the large mass of commensal bacteria. The local immunity in intestinal mucosa is basically ensured by gut associated lymphoid tissue (GALT), constituted by Peyer's patches, lymphoid follicles, and mesenteric lymph nodes [13]. Along with cellular, environmental, and genetic factors, deregulation of immune responses in the intestinal mucosa has been associated with the etiology of IBD. Alterations in the autophagy—a cellular process related to the degradation of intracellular pathogens, antigen processing, regulation of cell signaling and T cell homeostasis—usually results in reduced clearance of pathogens, thus contributing to the onset of inflammatory disorders in susceptible subjects [14, 15]. In this sense, mutations on ATG16L1 gene, a member of a family of genes involved in autophagy, were detected in patients with CD [16].

The breakage of self-antigens tolerance in the intestinal mucosa, by injury or genetic predisposition, may lead to CD or UC [17, 18]. Cells of the innate immunity, such as macrophages and dendritic cells, are specialized in identifying microorganism's molecular patterns by using the pattern recognition receptors (PRR), such as toll-like receptors (TLR) and nucleotide-binding oligomerization domains (NOD). In this regard, mutations in the caspase recruitment domain-containing protein 15 (CARD-15) gene encoding the NOD-2 protein were associated with the occurrence of IBD, especially CD. NOD2 is an intracellular microbial sensor that acts as a potent activator and regulator of inflammation. Therefore, deficiency in this protein promotes important changes on the immune response in the lamina propria, producing a chronic inflammation in the tissue. Clinically, it is of interest to determine the relationship between NOD2 gene status and the efficacy of antibiotic treatment in CD [19–22].

Likewise, the imbalance between Th1 and Th2 cytokines released by the intestinal mucosa determines the intensity and duration of the inflammatory response in experimental colitis [23]. The secretion of certain cytokines such as tumor necrosis factor-alpha (TNF-*α*) [24, 25], transforming growth factor-beta (TGF-*β*) [23, 26, 27], and interferon-gamma (IFN-*γ*) [28, 29] as well as the response to self-antigens [30–32] are factors that seem to be related to the onset and establishment of IBD. Although UC is often described as Th2-mediated diseases while CD is known as a Th1 condition, the classic paradigm has recently been changed, since cytokines can have diverse and opposing actions [33].

Recent data showed that Th17 cells and other cells producing interleukin- (IL-) 17 play a crucial role in the intestinal inflammatory manifestations. IL-17 and IL-22 appear to be related to the induction of colitis, since these cytokines initiate and amplify the local inflammatory signs and promote the activation of counter-regulatory mechanisms targeting intestinal epithelium cells [34]. Also IL-23, released by macrophages and dendritic cells located in the intestinal mucosa, activates signal transducer and activator of transcription- (STAT-) 4 in memory T lymphocytes, stimulating the production of IFN-*γ*. In turn, IFN-*γ* is responsible for triggering the production of inflammatory cytokines in cells of the innate immune system, contributing to the increase of the inflammation present in colitis [35]. Latest results from Neurath group [3] identified a pathogenic role of IL-9 in experimental and human ulcerative colitis by regulating intestinal epithelial cells.

It is also important to report that environmental factors can play a significant role in the development of IBD, although this relationship is poorly understood. Particularly, there are several evidences that tobacco could have an important role in triggering this type of intestinal inflammation [36, 37].

### 2.1. Crohn's Disease

Crohn's disease, one of the most frequent forms of inflammatory disease worldwide, is characterized by the formation of strictures, fistulas, ulcers, and granulomas in the mucosa. Although the CD's gastrointestinal manifestation can primarily affect the terminal ileum region, it can also compromise any region from the mouth to the rectum of affected patient. The clinical manifestations of CD can include diarrhea or bloody diarrhea, malnutrition, abdominal pain, and weight loss. Extraintestinal findings, for instance, arthropathy or skin disorders, rarely occur. However, manifestations on skin, muscle, or bone of metastatic Crohn's disease can actually lead to recognition of occult intestinal disease [80–82]. In general, CD has a genetic background and the first-degree relatives of affected individuals have a fivefold greater risk of developing the disease [83, 84].

The localized release of certain cytokines, such as IL-12, IL-17, TNF-*α*, and IFN-*γ*, has been implicated in the chronic intestinal inflammation observed in CD patients [51, 85]. The production of IL-12 and IL-18 by antigen-presenting cells (APC) and macrophages generates a polarized differentiation towards Th1 lymphocyte, leading to an increased release of proinflammatory cytokines, including TNF-*α* and IFN-*γ*. Additionally, Th1 cytokines stimulate the antigen-presenting cells to secrete a wider spectrum of inflammatory cytokines such as IL-1, IL-6, IL-8, IL-12, and IL-18, resulting in a self-sustained cycle [86].

### 2.2. Ulcerative Colitis

Ulcerative colitis is another form of IBD characterized by superficial ulcerations, granularity, and a vascular pattern. In contrast with the inflammation found in CD—transmural and being able to occur throughout the entire gastrointestinal tract—inflammation in UC is limited to the mucosal layer of the colon [87, 88]. Although Montreal classification—a system to classify IBD phenotypes including UC—is widely used, data on its reliability are very limited due to the great variety of clinical presentations of UC [88]. In general, the clinical manifestation of UC can include release of blood and mucus, petechial hemorrhage, and granulation tissue, among others. However, in periods of remission, the mucosa may have normal appearance. In most severe forms of the disease, the intestine can get distended, presenting deep ulceration and possibly intestinal perforation [87, 89].

In UC, there is a substantial increase in the secretion of IL-13, the main interleukin responsible for the inflammation and chronicity of this condition [87]. Despite the Th1 involvement, UC patients also present a Th2 response with increased secretion of IL-4, IL-5, and IL-9 [3, 5]. It has been suggested that the expression of the PU.1 transcription factor, a regulator of cellular communication, and the production of IL-9 by effectors' Th9 cells block the proliferation of intestinal epithelial cells and regulate the expression of several tight-junction proteins. Together, these aspects favor the translocation of specific bacterial species with subsequent activation of immune cells and mucosal inflammation in experimental and human UC [3]. As in CD, Th17-related cytokines are also increased in UC [34, 90].

## 3. Treatments for IBD

In order to better illustrate the relevance of each of the different IBD treatments, Table 1 compares different forms of treatment, mechanisms of action, patterns, and adverse effects of each form of therapy.

### 3.1. Classical Treatments for IBD

In ulcerative colitis, treatment decision is dependent on the stage of the disease: patients with mild manifestations are usually treated with aminosalicylates, whereas corticosteroids are prescribed for those with moderate disease and cyclosporine is given to patients with severe disease. In Crohn's disease, decisions about drug therapy are dependent on both location and behavior of the disease. Despite that, the medication in CD usually includes aminosalicylates and antibiotics to treat mild mucosal disease, corticosteroids to moderate disease, and biological molecules to treat fistulizing disease. Also, aminosalicylates, azathioprine, mercaptopurine, methotrexate, metronidazole, and associations can be used as maintenance therapies [38, 39, 42, 80, 91–94]. Notwithstanding their reduced cost, these drugs can generate several side effects. Moreover, these therapies do not achieve clinical remission and they can lead to the onset of other conditions such as renal impairment [39, 49, 50].

At the same time the classical treatments are widely used, new therapies are under development in the attempt of improving the patient's life quality. The new therapies aim to reduce the side effects and to treat patients who do not respond satisfactorily to conventional therapies [51, 52, 74, 95, 96].

Other therapeutic strategies, not covered in this review, are in very early evaluation. These involve the manipulation of the microbiome using antibiotics, probiotics, prebiotics, diet, and fecal microbiota transplantation [93, 97, 98].

### 3.2. Biological Therapies

The application of biological therapy for the treatment of inflammatory diseases can be associated with some studies that identified the proinflammatory cytokines present in the gut lamina propria of the IBD patients. These cytokines, in particular TNF-*α*, play a crucial role in the maintenance of chronic inflammation of the intestinal mucosa [53, 54]. Among the biological molecules, the use of monoclonal antibodies specific against TNF-*α*, cytokine related to the establishment of IBD, seems to be a relevant alternative. These antibodies may activate various mechanisms involved in the immune response, such as induction of apoptosis as well as the blockage of growth factors for the Th cells, antibody production, and complement activation [99].

Although the treatment of IBD with biological molecules, specifically with monoclonal antibodies, presents high specificity and directed mechanism of action, the high cost of this therapy still represents a barrier to be overcome. For this reason, together with this being a therapy in early stages of development, these drugs are generally used as an alternative for patients that are refractory to corticosteroid and aminosalicylates treatments [55, 100].

Also, long-term therapy with biological molecules can cause immunogenicity by generating anti-drug antibodies. These antibodies can promote acute and delayed infusion reactions and can reduce the duration of the patient response to each infusion or injection [101, 102]. In this sense, there is a potential contribution of the complement system as well as of the formation of immune complexes in the augmentation of immunogenicity [103, 104]. In some patients, immunogenicity is restricted to transient low level of antibodies, presenting no clinical effects. However, patients with high levels of anti-drug antibodies are more likely to present a loss of response by the reduction of drug levels, compromising the long-term therapy [101, 102]. As an alternative, concomitant immunosuppression appears to reduce immunogenicity and improve therapeutic control, even though it can present an increased risk for infection and malignancy [105].

Immunogenicity will depend on structure and origin of biologic agents. Biologicals can be fusion protein or a chimeric, and a humanized, or fully human antibody [106]. Also, the administration route, dosing schedule, and individual characteristics can have a great impact on immunogenicity [104, 106]. It is necessary to determine the optimal treatment regimen in order to minimize the likelihood of anti-drug antibody formation.

#### 3.2.1. Anticytokine Agents

Currently, some anticytokine agents have been showing relevant results for the treatment of IBD. It is already known that antibodies specific for TNF-*α* play an important role in maintaining the remission of CD, in both severe and moderate forms of the disease. These molecules were effective in inducing mucosal healing and clinical remission, reducing the cases of hospitalization and surgical procedures in affected individuals [52, 56, 57].

The first commercially available anti-TNF molecule was infliximab (IFX), a chimeric monoclonal IgG1 antibody [52, 56–58] formed by a segment of the native mouse protein containing the binding site for the TNF-*α* and a portion of human immunoglobulin responsible for the effector function of the antibody molecule [25, 54, 59]. With the introduction of IFX in market there was a considerable increase of clinical remission in CD and UC patients. In fact, IFX is currently the approved biologic agent for the treatment of inflammatory and fistulizing Crohn's disease and UC in several countries [52, 56, 57].

In order to treat nonresponsive patients to IFX, adalimumab (ADA), a fully human IgG1 monoclonal antibody, has emerged as an alternative molecule [58]. ADA is the first biotechnological product that is produced by using phage display technology approved by US Food and Drug Administration (FDA). Administration of ADA is indicated for individuals with CD in the moderate and severe forms, also showing positive results for the treatment of UC [58, 60]. The ADA induces apoptosis of proinflammatory cells by specifically binding to TNF-*α* molecule, leading to the blockage of the interaction of this cytokine with their surface receptors p55 and p75 [58].

Biological therapy with monoclonal antibodies IFX and ADA has been increasingly employed in the treatment of IBD, showing a significant improvement of the patient's clinical condition. In general, 45–70% of IBD cases presented clinical remission after being treated with IFX [43, 61, 107], while patients treated with ADA presented clinical remission near 30–60% [55, 61, 108].

However, the treatment with these molecules involves several risks and generates side effects. Renal complications, infusion reaction, delayed hypersensitivity-like reaction, new onset of autoimmunity (with rare cases of drug-induced lupus and new-onset demyelination) and opportunistic infections are some examples of complications resulting from the immunosuppression induced by biological therapy [25, 53, 54, 59, 62]. The fact that these drugs have been recently approved for clinical practice explains why their side effects are not thoroughly known. Because of that, more studies should be conducted in order to fully understand the mechanisms and the consequences of the use of these drugs. Furthermore, these anti-TNF agents did not present relevant effects in all treated patients, indicating another aspect worth investigating [55, 61, 109–111].

In this context, other anti-TNF biologic agents have emerged, including CDP 571, etanercept, and onercept. These humanized or fully human anti-TNF biotechnological agents are theoretically less immunogenic than the chimeric IFX. However, biopharmaceuticals such as etanercept (TNF inhibitor), onercept (recombinant TNF p55 receptor monomer), and CDP571 (recombinant humanized MAb against TNF-*α*) were not effective for active Crohn's disease [63, 112, 113].

Certolizumab pegol (CDP 870), a pegylated and fully humanized monoclonal antibody fragment, is an anti-TNF agent that has recently been approved by FDA for CD treatment with sustained remission [114–116]. Studies show that this drug seems to be more effective and less immunogenic than IFX and ADA [63, 64, 117]. Additionally, golimumab, a human IgG1 TNF-*α* antagonist monoclonal antibody, also showed significant results in inducing and maintaining remission in CD and UC patients, with a rate of adverse events similar to the placebo [63, 65, 118].

#### 3.2.2. Leukocyte Migration and Signaling Inhibitors

Since the application of biological drugs for treating IBD is a novel approach, there are several new biologic agents that have been recently approved or included in clinical trials or are under evaluation for determining their clinical efficacy and safety profile.

Currently, therapies that manipulate leukocyte adhesion, costimulatory signaling and cytokine receptors are being evaluated as potential treatments for IBD. These alternative treatments emerged when it was observed that some of the patients under current biologic therapies with anti-TNF-*α* agents were primarily nonresponders or experience a loss of response, intolerance, or even presented side effects [63, 75, 119–122].

Lymphocyte-endothelial interactions, mediated by adhesion molecules, are important in leukocyte migration and recruitment to sites of inflammation. The selective blockage of these adhesion molecules is a new and promising approach to treat CD. Recently approved by FDA, anti-*α*4 integrin monoclonal antibodies, specifically natalizumab and vedolizumab, were effective in the treatment of moderately to severely active CD (natalizumab and vedolizumab) and for UC (vedolizumab) patients [10, 63, 123]. The blockage of the T cell migration into the intestine by using anti-*α*4*β*7 antibody vedolizumab, approved to treat adult patients, resulted in a selective barrier for the trafficking of CD4+CD45RO+ T cells. It also reduced the UC clinical score, presenting a successful remission in 33% to 50% of cases [74, 76, 124].

Meanwhile, clinical efficacy of some therapeutic agents, such as inhibitors of leukocyte trafficking, including alicaforsen, an oligodeoxynucleotide that inhibits intercellular adhesion molecule 1 (ICAM-1) expression, are still under evaluation [123, 125–127]. Among the new drugs being tested, ustekinumab, a monoclonal antibody against the p40 subunit of interleukin-12/23, approved to use in patients with moderate or severe psoriasis and psoriatic arthritis, was able to induce clinical response in patients with moderate-to-severe CD, especially in those previously treated with IFX [51, 66]. Etrolizumab, a humanized monoclonal antibody that selectively binds to the *β*7 subunit of the heterodimeric integrins *α*4*β*7 and *α*E*β*7, was well tolerated in moderate to severe UC on phase II studies [128, 129]. Additionally, tofacitinib, a small molecule targeting Janus-activated kinase (JAK), was shown to particularly inhibit JAK1 and JAK3, also interfering with several cytokine receptors. However, there are no relevant clinical data related to this molecule [130].

Since one of the most important mechanisms in establishing gastrointestinal inflammatory conditions is the activation of different populations of T cells, the balance among effectors and regulatory populations is crucial for driving the immune response in GALT [131]. In this way, costimulatory signaling of T cell activation has been investigated as a potential target to block unwanted and deleterious inflammatory response. One of these targets is the CTLA-4 molecule, expressed on the surface of T cells, which selectively competes with CD28 molecule for binding to CD80 and CD86 molecules present on APCs. Besides that, it was recently demonstrated that both T regulatory and T conventional cells exert a suppressive function on the externalization of CTLA-4 protein [132]. Thus, new clinical approaches have used the biological molecule abatacept, a fusion protein composed of the Fc portion of IgG together with the CTLA4 molecule (CTLA4-Ig), to treat different inflammatory disorders such as psoriatic arthritis, type 1 diabetes, multiple sclerosis, and systemic lupus erythematosus. However, a phase III trial in moderate-to-severe CD and UC showed no therapeutic benefits with the use of the abatacept, indicating that blocking the T cell activation possibly compromises the activation of important regulatory T cells subsets in IBD patients [133].

#### 3.2.3. Biological Drug Dosage

Nowadays, five biologic agents are approved by FDA for the treatment of IBD: adalimumab, infliximab, golimumab, certolizumab pegol, and vedolizumab. In order to reach an effective disease remission of IBD patients, IFX standard dosage for UC and CD is usually 5 mg/kg by intravenous infusion at weeks 0, 2, and 6, followed by a maintenance regimen every 8 weeks. However, some data shows that the dosage of 10 mg/kg seems to maintain the remission for a longer period [134–137]. On the other hand, ADA has shown to be effective to UC and CD by the subcutaneous administration with an initial dose of 160 milligrams, a second dose two weeks later of 80 mg, and a maintenance dose of 40 mg every other week, although it has also been shown that there is a dose-dependent effectiveness related to this drug [138–140].

In patients with moderate to severe CD, subcutaneous administration of certolizumab pegol on subcutaneous doses of 400 mg once every 4 weeks was effective as induction and maintenance therapy. In case of lack of response, it should be given every 2 weeks [141]. The recommended golimumab initial regimen for the treatment of UC is a 200 mg subcutaneous dose at week 0 followed by 100 mg at week 2. The maintenance therapy is 100 mg every 4 weeks [67]. Vedolizumab was recently approved by FDA for the treatment of adults with moderately to severely active UC and CD. Dose regimen is 300 mg infused intravenously at 0, 2, and 6 weeks and the maintenance therapy at every 8 weeks thereafter [74].

Despite the fact that there are no suficient comparative trial data available between infliximab, adalimumab, and certolizumab pegol, they are considered as having comparable efficacy, especially when the maintenance of remission is taking into consideration [68, 137]. An advantage of ADA, golimumab, and certolizumab in comparison with IFX and vedolizumab is that they can be administered by a subcutaneous injection. It is important to mention that patient's history, drug regimen, and drug efficacy create a singular scenario that should be taken into account before choosing the appropriate therapy.

#### 3.2.4. Biosimilars

Additionally, it is important to mention that infliximab's patent has already expired in many countries (e.g., Brazil, Argentina, Canada, South Korea, and some Eastern Europe countries) and is about to in USA and in Western Europe countries, opening the opportunities for biosimilar drugs to reach the market. Nowadays, IFX-biosimilars are already used in some countries for the treatment of IBD and present a significant reduction in costs.

Biosimilars present the same amino acid sequence and a highly similar glycosylation pattern when compared with the original product. Accordingly, biosimilars represent a future tendency and a promising new option for IBD patients with the main advantage of being less expensive, which may affect the availability of the biological treatment for patients around the world. However, concerns about the efficacy, safety, and immunogenicity of biosimilars still exists [142, 143].

## 4. Final Considerations

Gastrointestinal immune disorders are most likely facilitated by defects in the intestinal epithelial barrier and in the mucosal immune system, resulting in an active inflammation and tissue destruction. The mucosal immune system is essential for the establishment and controlling of the intestinal inflammation and injury, with cytokines playing a central role in modulating IBD. Therefore, using specific inhibitors or blockers targeting cytokines and chemokines may be a strategic move for treating IBD. Essentially, approved biological molecules for commercial use act as specific inhibitors of inflammatory cytokines related to autoimmune diseases. Among approved TNF blockers, IFX and ADA are the most commonly used biological drugs for the treatment of IBD. Despite the fact that half of the patients treated with these biological molecules have shown clinical remission and its clinical benefits seemed to outweigh the risks involved, there is a growing concern regarding the development of immunogenicity against the biologics, since some patients may develop anti-drug antibodies.

Albeit many studies are still ongoing with the goal of using biological therapy, the effective cost of its production is very elevated in comparison with other drugs, which might make this a hard to implement treatment. Furthermore, treatment with biologicals must be defined carefully, since several drugs need further preclinical and clinical studies prior for their use to be considered as a first option treatment. In this sense, more controlled clinical trials are currently being conducted, exploring the safety and efficacy of old and new biologic agents. Regardless, the most recently engineered biological drugs will certainly open a fresh and exciting perspective on the development and improvement of therapies for IBD.

## Figures and Tables

**Table 1:** IBD treatments: drugs in use, mechanisms of action, and side effects.

Treatment type	Related drugs	Mechanism of action	Features	Potentials adverse effects	References
Aminosalicylates	Mesalamine Olsalazine Balsalazide Sulfasalazine	Inhibition of IL-1, TNF-*α*, and platelet activating factor (PAF), decreased antibody secretion.	Locally immunosuppressive, nonspecific inhibition of cytokines; medium cost.	Headache, dizziness, dyspepsia, epigastric pain, abdominal pain, nausea, vomiting, and diarrhea.	[38–41]

Immunomodulators	Azathioprin 6-mercaptopurin Methotrexate	Blockage of *de novo* pathway of purine synthesis.	Antiproliferative effects, reduction of inflammation.	Black, tarry stools, bleeding gums, chest pain, fever, chills, swollen glands, pain, cough, and weakness.	[42–48]

Corticosteroids	Prednisone Methylprednisolone Hydrocortisone Budesonide	Blockage of phospholipase A2 in the arachidonic acid cascade altering the balance between prostaglandins and leukotrienes; stimulation of apoptosis of lamina propria lymphocytes; suppression of the transcription of cytokines.	High immunosuppression, risk of potential infections, adverse effects with long periods of use, low cost.	Full moon face, difficulty of healing, acne, sleep and mood disturbances, glucose intolerance, osteoporosis, osteonecrosis, subcapsular cataracts, myopathy, infections, acute adrenal insufficiency, myalgia, malaise, arthralgia or intracranial hypertension, and pseudorreumatism syndrome.	[49, 50]

Biologicals: anti- cytokine drugs	Infliximab Adalimumab Certolizumab-pegol Golimumab Ustekinumab (phase 3 trial)	Induction of apoptosis in proinflammatory cells; binding specifically to TNF-*α*, blockage of the interaction the receptor.	Specific inhibition of cytokine, immunosuppression, high cost, advanced technology required.	Abdominal or stomach pain, chest pain, chills, cough, dizziness, fainting, headache, itching, muscle pain, nasal congestion, nausea, sneezing, weakness, vomiting, bloody urine, cracks in the skin, diarrhea, pain, fever, abscess, back or side pain, bone or joint pain, constipation, falls, facial edema, general feeling of illness, hernia, irregular heartbeat, unusual bleeding, weight loss, increased risk of reactivation of latent tuberculosis, and increased risk for developing infections and lymphoma.	[24, 51–73]

Biologicals: anti-cell adhesion molecule	Vedolizumab Natalizumab	Inhibition of migration.	Specific inhibition of cell adhesion molecules high cost, advanced technology required.	Nasopharyngitis, headache and abdominal pain, increased risk of infections, serious infections, and progressive multifocal leukoencephalopathy (natalizumab).	[63, 74–79]
